# Effect of BLV Infection on the Immune Function of Polymorphonuclear Neutrophil in Dairy Cows

**DOI:** 10.3389/fvets.2021.737608

**Published:** 2021-09-23

**Authors:** Guanxin Lv, Hai Wang, Jianfa Wang, Shuai Lian, Rui Wu

**Affiliations:** ^1^College of Animal Science and Veterinary Medicine, Heilongjiang Bayi Agricultural University, Daqing, China; ^2^Heilongjiang Provincial Key Laboratory of Prevention and Control of Bovine Diseases, Daqing, China

**Keywords:** bovine, bovine leukemia virus, neutrophils, bactericidal function, immunosuppression

## Abstract

Enzootic bovine leukemia is a late-onset, neoplastic infection caused by the bovine leukemia virus (BLV). BLV infection hinders the function of the immune system and induces other diseases, which negatively affects the performance and health of the infected cows. As the first line of defense against invading foreign pathogenic microorganisms, polymorphonuclear neutrophil (PMN) plays a vital role in the immune system of dairy cows. However, research on the effect of BLV infection on the immune function of PMN in dairy cows is scarce. Therefore, this experiment aimed to elucidate the effects and effect mechanisms of BLV infection on the immune function of PMN in dairy cows with different BLV provirus loads by detecting the chemotaxis, migration, adhesion, phagocytosis, respiratory burst function, and the formation of NETs. The experimental results showed that BLV infection had no significant effect on the phagocytosis of PMN but inhibited their migration and respiratory burst function, and the effects were closely related to the BLV provirus load. Under high BLV provirus load, PMN produced large amounts of NETs, chemokine CXCL7, adhesion molecule CD18, and pro-inflammatory factors IL-8 and TNF-α, triggering inflammatory responses, and tissue damage. The results of this study will help reveal the reason why BLV infection causes the high incidence of mammary gland inflammation in dairy cows.

## Introduction

Bovine leukemia virus (BLV), a virus of the Retroviridae family, Deltaretrovirus genus, is closely related to human T-lymphocyte leukemia virus 1 and 2 (HTLV-1, HTLV-2). BLV causes Enzootic Bovine Leukemia (EBL), a disease that has caused widespread epidemics and substantial economic losses to dairy farming worldwide since first discovered in 1871 (1). To date, BLV infection and transmission are still prevalent in North America and Asia. Only 21 countries worldwide have eradicated BLV, most of which are located in Western Europe (2, 3). Typically, BLV causes persistent subclinical infection, with most infected cattle showing no obvious clinical signs, about 30% developing persistent lymphocytosis (PL), and about 5% dying of malignant lymphoma (4). BLV mainly infects peripheral blood lymphocytes and monocytes, leading to diminished immune system function, increased susceptibility to multiple infections, and increased disease severity, ultimately affecting milk production, milk quality, and cow culling rates (5, 6).

A retrovirus could integrate its genome into the host genome and exist as a provirus. The proviral load (PVL) could help predict virus-associated diseases and the transmission risk. For example, the PVL of BLV-infected cattle in the lymphoma stage is significantly higher than those in the PL stage and aleukemic (AL) stage. Research on BLV defines high PVL (H-PVL) and low PVL (L-PVL) with a threshold of 1,000 copies/10 ng DNA (7). Studies have reported that 65% of the cows with EBL have H-PVL and are more likely to infect other cows (8, 9). In addition, the severity of mastitis in cows is related to the peripheral blood BLV PVL. H-PVL cows have reduced mammary immune function compared to L-PVL cows (10). It is hypothesized that BLV infection may cause changes in the natural immune characteristics of dairy cows, which may affect the function of their immune system, increase bacterial susceptibility, and influence the development and prognosis of diseases (11–13).

Polymorphonuclear Neutrophil (PMN) plays a vital role in the natural immune system and is generally in dynamic equilibrium with a short life span and essentially constant numbers in circulation. Under a pathological state, a large amount of PMN is produced to resist the invasion of pathogenic microorganisms. Induced by inflammatory factors and chemokines, activated PMNs travel rapidly with blood flow to the endothelial cells near the inflammation and express β2 integrins CD11b and CD18, which bind tightly to the adhesion molecules ICAM-1 and ICAM-2 on the surface of endothelial cells. PMNs then enter the site of infection by transendothelial migration and kill the invading pathogenic microorganisms by phagocytosis, respiratory burst, and formation of neutrophil extracellular traps (NETs). In addition, PMNs also produce cytokines and other inflammatory factors that regulate the entire immune system (14). Thus, PMNs are also vital effector cells of the acquired immune system. However, when PMN exerts its immune function, the excessive NETs and superoxides released by respiratory burst could lead to oxidative stress and exacerbate the inflammatory response, causing organismal damage. Therefore, PMN has both favorable and unfavorable effects on the development of disease (15, 16).

At present, studies have shown that PMN is the first immune cell recruited to the site of infection after the virus invades. It mediates the antiviral immune response by interacting with other immune cell groups to produce chemokines, cytokines, ROS and release NETs. Blagitz et al. found that the percentage of neutrophils that phagocytosed *Staphylococcus aureus* was lower in BLV-infected dairy cows. The relative percentage of CD44+ neutrophils and CD11b expression by neutrophils was also lower in BLV-infected dairy cows. The percentage of neutrophils producing ROS was lower in BLV-infected cows. Furthermore, the percentage of CD44+ monocytes was positively correlated with the percentage of neutrophils that phagocytosed S. aureus. In BLV-infected dairy cows, inhibition of neutrophil apoptosis was observed. (17). The study by Della Libera et al. (11) showed that PMN and B lymphocytes in BLV-positive cow milk would also be similarly affected. Currently, all the studies were studied in lymphocyte proliferation in naturally BLV-infected dairy cows, PL and non-PL animals (18, 19). No study has regarded the different BLV proviral load that may bias the previous findings, but the study by Juliarena et al. (20) showed that a considerable proportion of AL cows showed the same H-PVL as PL animals.

The specific mechanisms by which BLV affects the immune system function are unclear. Few studies focused on the effects of BLV infection on the immune function of PMNs, the primary immune cells involved in innate immunity, in cows. Therefore, this experiment studied the correlation between BLV and the immune function of PMNs in dairy cows and, for the first time, set different BLV PVL groups to explore the effects on PMN chemotaxis, migration, adhesion, phagocytosis, respiratory burst, and the formation of NETs. This study may provide an experimental basis for scientific prevention and control of BLV infection and maintenance of dairy cow immune system health.

## Materials and Methods

### Experimental Design and Sample Collection

The test animals were selected from a sizeable intensive dairy farm in Daqing, Heilongjiang Province, China. A total of 107 healthy cows of similar age, body condition, and lactation were randomly selected. Whole blood was collected from the cows *via* the middle tail vein. A portion of the whole blood was used for serum separation, and the serum samples were tested using the IDEXX Bovine Leukemia Blocking Antibody Test Kit® (IDEXX, USA) according to the manufacturer's instructions. The other portion of the whole blood sample was used for DNA extraction with the TIANGEN Blood/Cell/Tissue Genomic DNA Extraction Kit® (Tiangen, Bejing, China) according to the manufacturer's instructions. Refer to the detection method of quantitative detection of BLV previrus in OIE laboratory (21, 22). The plasmid standard of BLV full-length sequence kept in our laboratory was used as the template. The plasmid concentration was measured as 570 ng/μL using a micro UV-Vis spectrophotometer. The plasmid copy number was calculated according to the copy number formula as 4.17 × 10^10^ copies/μL.


(1) $$\begin{array}{l} copies/\mu L=\left(6.02\times 10^{23}\right)\\ \;\times \left(\text{ng}/\mu L\times 10^{-9}/\left(DNA\right)L\text{ength}\times 660\right) \end{array}$$

The plasmid was then diluted to the concentrations ranging from 4.17 × 10^8^ to 41.7 copies/μL. Absolute quantitative PCR amplification was performed in a fluorescent qPCR instrument according to the TaKaRa Probe qPCR Mix operating instructions (TaKaRa, Dalian, China), and standard curves were plotted. The primers and probe sequences are listed in Table 1 (23). Quantitative PCR was carried out with the CFX96 systems using a final reaction volume of 25 μL (Table 2). After successfully constructing the standard curves, the extracted DNA samples were subjected to absolute quantitative PCR using the above method and reaction conditions. The viral copy number was calculated using Equation (1). Viral copy number above 1,000 copies/10 ng DNA was defined as H-PVL, and vice versa as L-PVL. According to the ELISA and absolute quantitative PCR results, the test animals were divided into the healthy group, the L-PVL group, and the H-PVL group. Five cows from each group were selected for the subsequent testing.

**Table 1:** Sequences of the primers used for detection of cytokine.

**Gene**	**Primer sequence**	**Product size**
**pol**	Forward: 5′-CCTCAATTCCCTTTAAACTA-3′	120 bp
	Reverse: 5′-GTACCGGGAAGACTGGATTA-3′	
	Probe: FAM-GAACGCCTCCAGGCCTTCA-BHQ	

**Table 2:** The protocol in qRT-PCR reaction.

**Gene**	**Initial denaturation**	**Subsequent denaturation**	**Annealing**
pol	95°C 30 s 1×	95°C 5 s 40×	60°C 30 s

### PMN Isolation and Identification

Blood samples were collected from cows of the healthy group, the L-PVL group, and the H-PVL group. PMNs were isolated using the Solarbio Bovine Peripheral Blood Neutrophil Isolation Kit® (Solarbio, Bejing, China). Then, 4 mL Separate A, 2 mL Separate C, and 2 mL sodium citrate anticoagulated whole blood were successively added into a 15 mL centrifuge tube, forming a liquid gradient. The mixture was then centrifuged at room temperature in an Eppendorf high-speed centrifuge at 900 × g for 30 min (Eppendorf, Germany). After centrifugation, the mixture was stratified. The PMNs in the white ring-like cell layer near the bottom were transferred to a new 15 mL centrifuge tube. The volume ratio of neutrophil suspension to erythrocyte lysate was 1:3, and an appropriate amount of red blood cell lysis buffer was added to the centrifuge tube to lyse the remaining red blood cells. The mixture was centrifuged at 500 × g for 10 min, and the supernatant was discarded. The above steps were repeated several times until there was no apparent red color in the cell precipitate. Subsequently, 3–5 mL cell washing solution was added to repeatedly wash the cells while centrifuging at 500 × g for 5 min each time. The obtained cell precipitates were resuspended to the desired densities with HBSS buffer, RPMI-1640 medium, or RPMI-1640 medium (Gibco, Grand Island, NY, USA) containing 10% FBS (Gibco Grand Island, NY, USA) according to the different experimental needs.

The obtained PMNs were stained with DAPI and observed under a laser confocal microscope to identify the cell morphology. Take 10 μL of cell suspension and trypan blue staining solution respectively, mix them evenly, incubate for 30 s, and drop 10 μL of mixed solution into the automatic cell counting plate, and their concentration and viability were measured using an automated cell counter.

### Chemotaxis and Migration Assay

PMNs were resuspended with HBSS buffer to a cell density of 2 × 10^6^ cells/mL and repeatedly freeze-thawed before centrifuged at 3,000 r/min for 20 min. The supernatant was collected. The secretion of chemotactic protein NAP-2/CXCL7 was measured using the Lengton Bovine NAP-2/CXCL7 ELISA Kit® (Lengton, Shanghai, China) following the manufacturer's instructions.

PMNs were resuspended with RPMI-1640 medium to a cell density of 1×10^6^ cells/mL. Then, 200 μL cell suspension was added into the upper chamber of Transwell chambers (3.0 μm, 24-well-insert; Corning, Lowell, MA, USA), and 400 μL RPMI-1640 medium containing 10% FBS was added into the lower chamber. The Transwell chambers were placed in an incubator with 5% CO_2_ and incubated at 37°C for 3 h. The number of PMNs migrated to the lower chamber was measured with flow cytometry (Beckman Coulter, CA, USA).

### Phagocytosis Assay

PMNs and FITC-labeled *S. aureus* were placed in an incubator with 5% CO_2_ and coincubated at 37°C for 1 h. The mixture was then placed into an icebox to terminate phagocytosis. After centrifuged at 4°C, 1,000 r/min for 5 min, the supernatant was discarded. The precipitate was washed twice with PBS buffer. The fluorescence intensity of the PMNs was measured using flow cytometry.

The percentage of PMNL that phagocytized the bacteria was calculated as the number of fluorescent PMNL divided by the total PMNL count multiplied by 100. The median fluorescence intensity of phagocytosis was estimated by the geometric median of the FITC fluo-rescence/PMNL with phagocytized bacteria. For this assay, 10,000 gated neutrophils cells were examined in each sample. The Flow Jo Tree Star Software (TreeStar Inc.) was used to analyze the data (24).

### NETs Assay

Polylysine-treated round coverslips were placed in a 24-well-plate. PMN suspension was added into the wells with a density of 2.5 × 10^5^ cells/well. Phorbol 12-myristate 13-acetate (PMA) was added to the wells. The final concentration of PMA was adjusted to 100 nmol/L. The well-plate was placed in an incubator with 5% CO_2_ and incubated at 37°C for 3 h. Then, 4% paraformaldehyde was added to fix the cells at room temperature for 1 h. The cells were then washed three times with PBS-T wash buffer. Subsequently, 0.3% Triton X-100 was added to permeabilize for 15 min before blocking in 10% goat serum for 1 h. The cells were incubated in a refrigerator at 4°C overnight with PBS buffer diluted mouse-derived MPO and rabbit-derived ELA2 (Proteintech Group, Wuhan, USA). The cells were washed three times with PBS-T wash buffer and incubated in the dark for 1 h after adding Alexa Fluor® 488-labeled goat anti-mouse IgG and Alexa Fluor® 594-labeled goat anti-rabbit IgG (ZSGB-BIO, Bejing, China). The cells were again washed three times. The anti-fluorescence quenching sealing agent containing DAPI (Solarbio, Bejing, China) was added dropwise to the round coverslips, which were then removed from the well-plate and inverted on slides. The cells were visualized under a laser confocal microscope (Leica, Germany).

### Respiratory Burst Assay

The respiratory burst function of PMNs was measured using the Absin Neutrophil Respiratory Burst Assay Kit® (Absin, Shanghai, China). Briefly, dihydrorhodamine 123 was added to the whole blood. The mixture was incubated in the dark for 5 min at 37°C in a constant temperature incubator. Then, a 10-fold dilution of hemolysin was added to hemolysate in the dark at room temperature for 15 min. The mixture was centrifuged for 5 min at 1,500 r/min. The supernatant was discarded, and the precipitate was washed twice with PBS buffer. Flow cytometry was used to measure the results.

The percentage of neutrophils producing ROS was calculated as the number of fluorescent neutrophils divided by the total neutrophil count and multiplied by 100. The median fluorescence intensity (MFI) of ROS production was estimated from the median of Rh123 fluorescence divided by the number of neutrophil that produced ROS. For this assay, 10,000 gated neutrophils were examined per sample. The FlowJo software (TreeStar Inc., Ashland, USA) was used to analyze the data. The results were corrected for autofluorescence content, which was defined as the fluorescence that was associated with the non-labeled freshly isolated milk cells from the same cow (24).

### Cytokine Assay

PMN RNA was extracted using the TIANGEN Total RNA Extraction Kit® (Tiangen, Bejing, China) before reverse transcriptase using the PrimeScript™ RT reagent Kit with gDNA Eraser (TaKaRa, Dalian, China) according to the manufacturer's instructions. Then, the expression of CD11b, CD18, IL-8, and TNF-α mRNAs in PMN was examined using the SYBR® Premix Ex Taq (TaKaRa, Dalian, China) according to the manufacturer's instructions. The primers are listed in Table 3, and the qRT-PCR reaction procedures are listed in Table 4.

**Table 3:** Sequences of the primers used for detection of cytokine.

**Gene**	**Primer sequence**
CD11b	Forward: 5′-CAAACTGGCAGAAAGCAACA-3′
	Reverse: 5′-TCCAGGAAGACTCTGGAGGA-3′
CD18	Forward: 5′-AGCGACCTCAGGGAGTACCAT-3′
	Reverse: 5′-GTCGTGGTGGCACTCTTGAAA-3′
IL-8	Forward: 5′-GCTGGCTGTTGCTCTCTTG-3′
	Reverse: 5′-GGGTGGAAAGGTGTGGAATG-3′
TNF-α	Forward: 5′-CTGGCGGAGGAGGTGCTCTC-3′
	Reverse: 5′-GGAGGAAGGAGAAGAGGCTGAGG-3′
GAPDH	Forward: 5′-GGCATCGTGGAGGGACTTATG-3′
	Reverse: 5′-GCCAGTGAGCTTCCCGTTGAG-3′

**Table 4:** The protocol in qRT-PCR reaction.

**Gene**	**Initial denaturation**	**Subsequent denaturation**	**Annealing**	**Extension**
pol	95°C 30 s 1×	95°C 5 s 40×	60°C 30 s 40×	95°C 10 s 40×

### Statistical Analysis

Data were analyzed using GraphPad Prism 8.0 software®(GraphPad Software, Inc., San Diego, CA, USA). One-way ANOVA was used to compare the groups. The data obtained were expressed as mean ± SEM. *P* < 0.05 was considered statistically significant.

## Results

### BLV Infection Status and Proviral Load Detection

ELISA testing was performed on the 107 cows to detect BLV antibodies, and 23 were found positive, showing a positivity rate of 21.5%. PVL of the cows tested positive were analyzed using qPCR, and 5 cows were found with H-PVL, accounting for 21.7% of the positive cows (Table 5). The test animals were then divided into the healthy group, the L-PVL group, and the H-PVL group based on the results above, with 5 cows in each group for subsequent experiments. Dynamic monitoring of the proviral load of the selected test animals during the test period. The results showed that the PVL in the L-PLV group did not change significantly, while the PVL of H-PVL group were increased to varying degrees. There is no effect on the grouping of experimental animals.

**Table 5:** Copy number of BLV positive bovine provirus.

**Number**	**Bovine number**	**Number of pre BLV copies (copies/10 ng DNA)**
(1)	92956	1,699.8
(2)	6469	1,181.8
(3)	86042	1,178.4
(4)	6507	1,122.1
(5)	18169	1,016.8
(6)	17004	650.5
(7)	92259	199.2
(8)	102652	93.7
(9)	18039	91.1
(10)	92101	77.2
(11)	17003	40.8
(12)	92079	28.1
(13)	6556	20.8
(14)	92998	19.0
(15)	92197	12.9
(16)	100479	11.9
(17)	160505	10.5
(18)	100294	6.9
(19)	0517	2.2
(20)	88597	2.0
(21)	88252	1.5
(22)	6512	1.3
(23)	6543	0.5

### PMN Isolation and Identification

**Figure 1:**
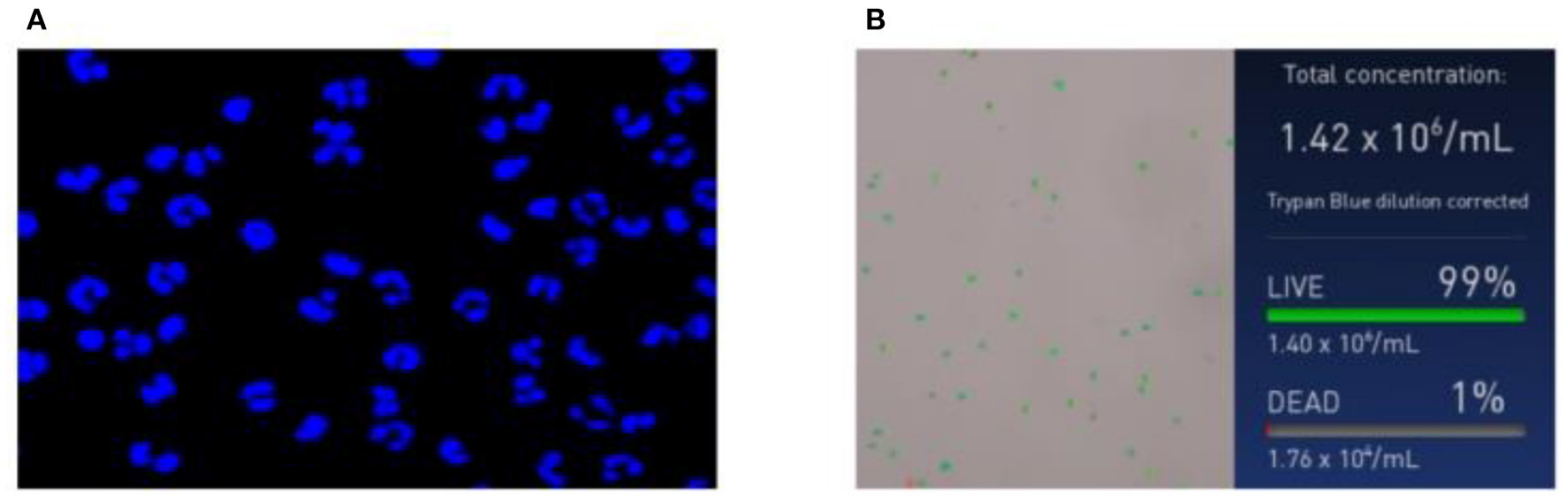
Neutrophil identification results. **(A)** Morphological images of neutrophils and **(B)** concentration and survival rate images of neutrophils.

The isolated PMNs were DPAI and observed *via* laser confocal microscopy. As shown in Figure 1A, PMNs have a unique lobed nucleus structure, and the purity is above 90%. The number and viability of the isolated PMNs were counted using an automated cell counter (Figure 1B). The PMN number and activity met the requirements of subsequent experiments. All experiments in this paper were based on this condition.

### Effect of BLV on PMN Chemotaxis and Migration Function

**Figure 2:**
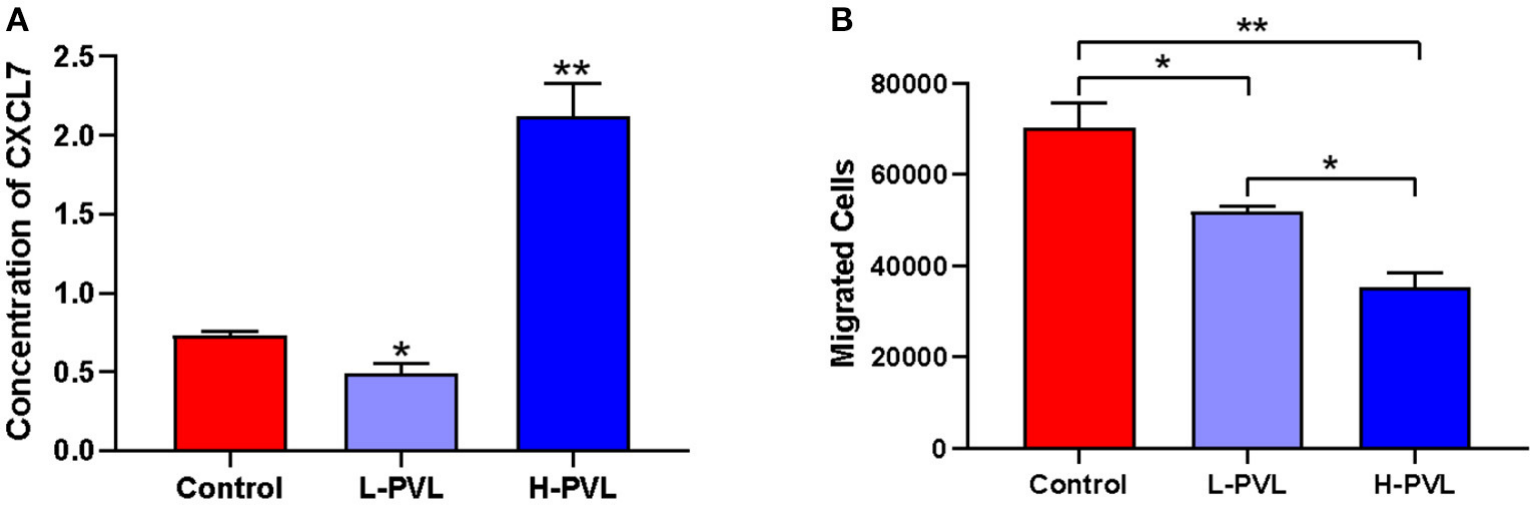
Effect of BLV infection on chemotaxis and migration of PMN. **(A)** Effect of BLV infection on chemotaxis of PMN and **(B)** Effect of BLV infection on migration function of PMN. *P < 0.05 compared to healthy cows, **P < 0.01 indicates significant difference compared to healthy cows.

The secretion of chemotactic protein NAP-2/CXCL7 in cows of the healthy group, the L-PVL group, and the H-PVL group was measured with the NAP2/CXCL7 ELISA kit to investigate the effect of different BLV PVL on the chemotactic function of PMN in cows (Figure 2A). The expression of CXCL7 was significantly lower in cows of the L-PVL group compared to cows of the healthy group, but the expression CXCL7 was significantly higher in cows of the H-PVL group (*P* < 0.05).

The number of PMNs migrated to the lower chamber of Transwell chambers was counted using flow cytometry to reveal the effect of different BLV PVL on the migration function of PMN in cows (Figure 2B). The number of migrated PMNs was significantly lower in cows of the L-PVL and H-PVL groups compared to cows in the healthy group (*P* < 0.05), and the number of migrated PMNs was significantly lower in cows of the H-PVL group compared to cows of the L-PVL group (*P* < 0.05).

### Effect of BLV on PMN Phagocytosis

**Figure 3:**
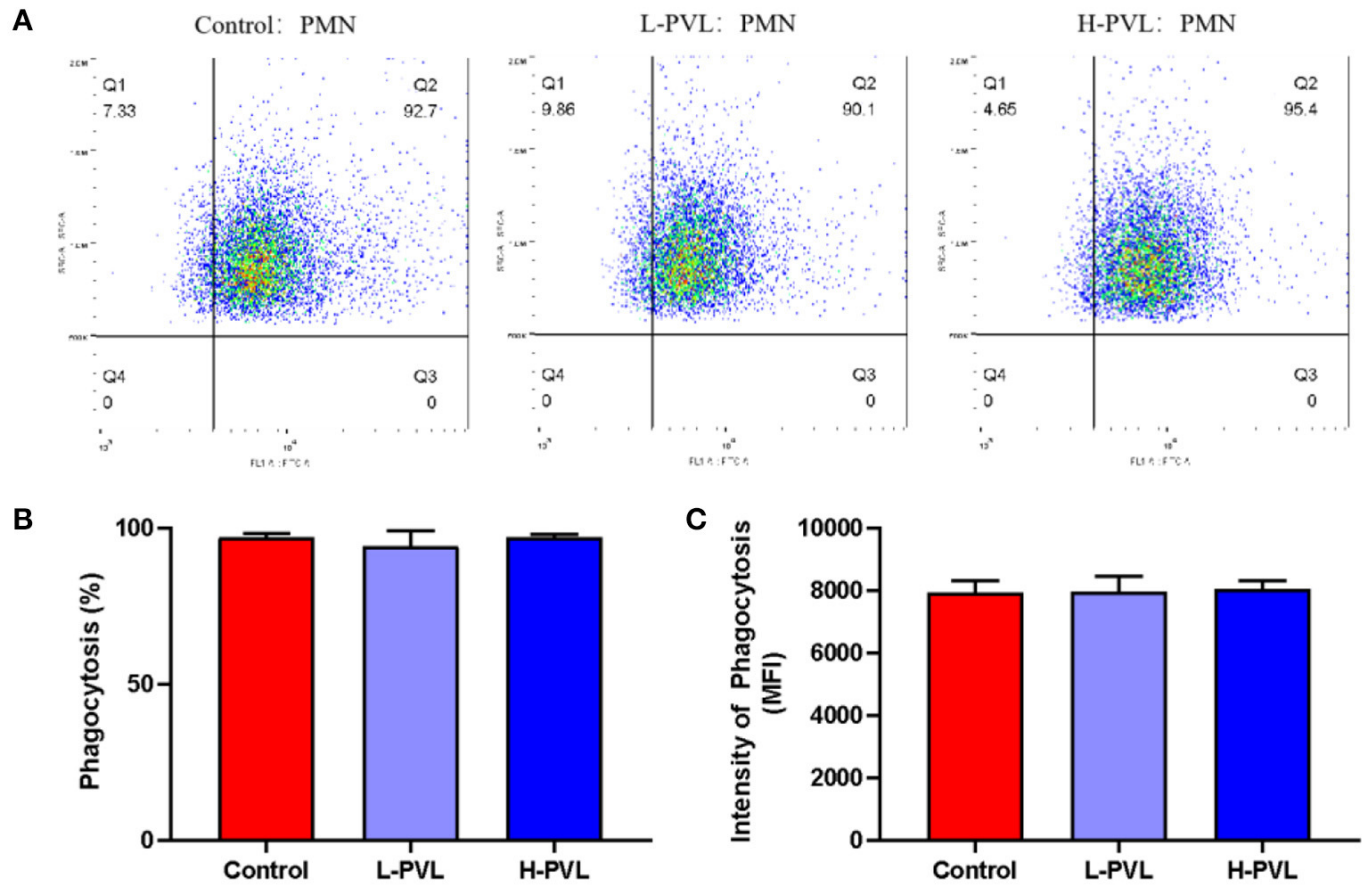
Effect of BLV infection on phagocytosis of PMN. **(A)** Effect of BLV infection on phagocytosis of PMN, **(B)** Effect of BLV infection on phagocytic efficiency of PMN, and **(C)** Effect of BLV infection on phagocytosis of PMN.

PMNs and FITC-labeled *S. aureus* were co-incubated, and the MFI was measured using flow cytometry to reflect the changes in the phagocytic capacity of PMN and reveal the effect of different BLV PVL on the phagocytosis of PMN in cows (Figure 3). There was no significant change in phagocytosis efficiency (Figure 3B) or phagocytosis intensity (Figure 3C) in cows of the L-PVL and H-PVL groups compared to cows of the healthy group (*P* > 0.05).

### Effect of BLV on PMN NETs Formation

**Figure 4:**
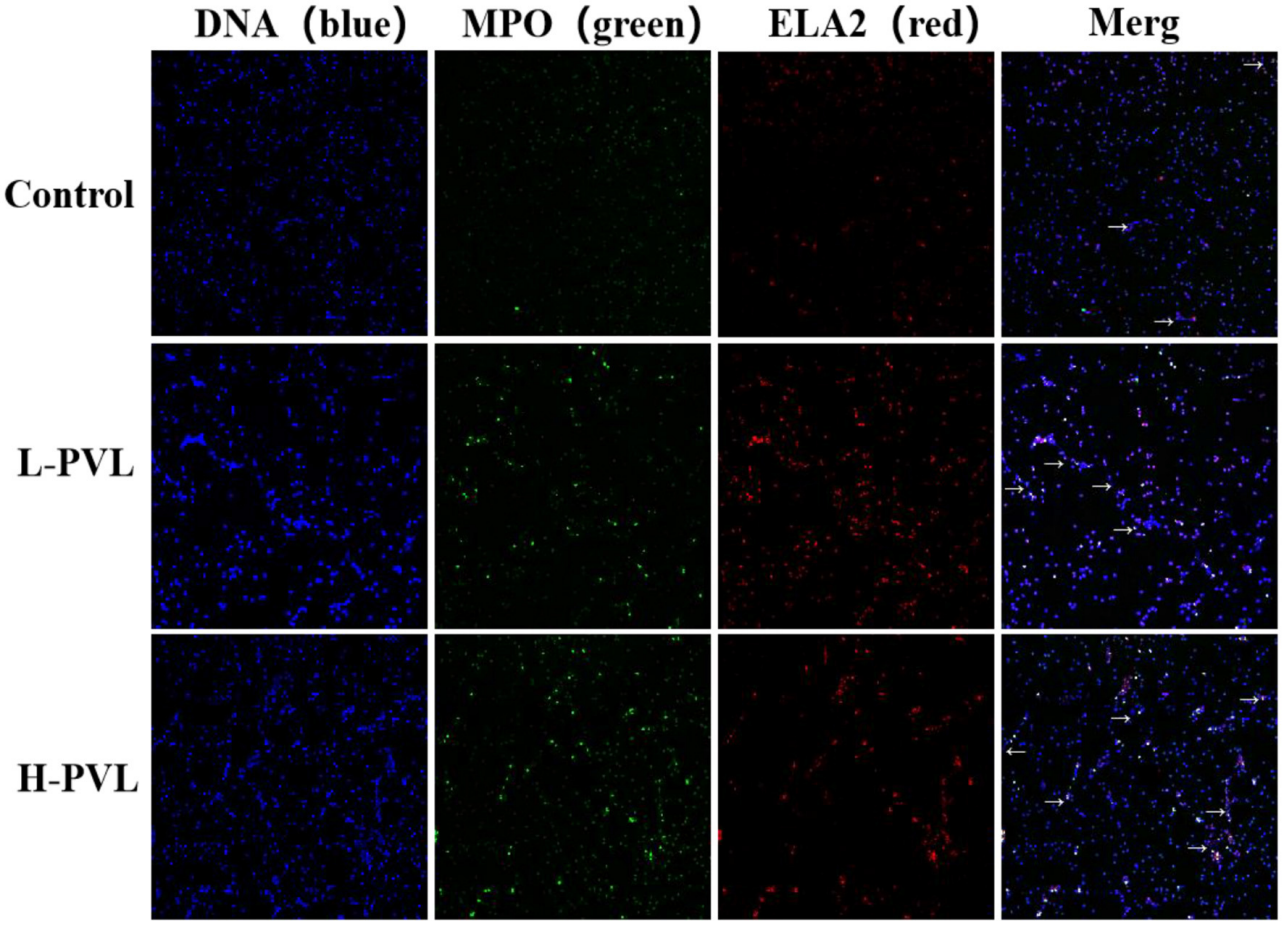
Effect of BLV infection on the formation of PMN NETs (100×).

PMA was used to stimulate PMNs to produce NETs, and the DNA backbone, MPO, and ELA2 were examined in healthy, L-PVL, and H-PVL cows to reflect the formed NETs and reveal the effect of different BLV PVL on the formation of NETs in cows. The results are shown in Figure 4, where the NETs are pointed out with white arrows. There was no significant change in the formation of NETs in cows of the L-PVL group compared to cows of the healthy group (*P* > 0.05), but there was a significant increase in the formation of NETs in cows of the H-PVL group (*P* < 0.05. There was no significant change in the formation of NETs in cows of the H-PVL group compared to cows of the L-PVL group (*P* > 0.05).

### Effect of BLV on PMN Respiratory Burst

**Figure 5:**
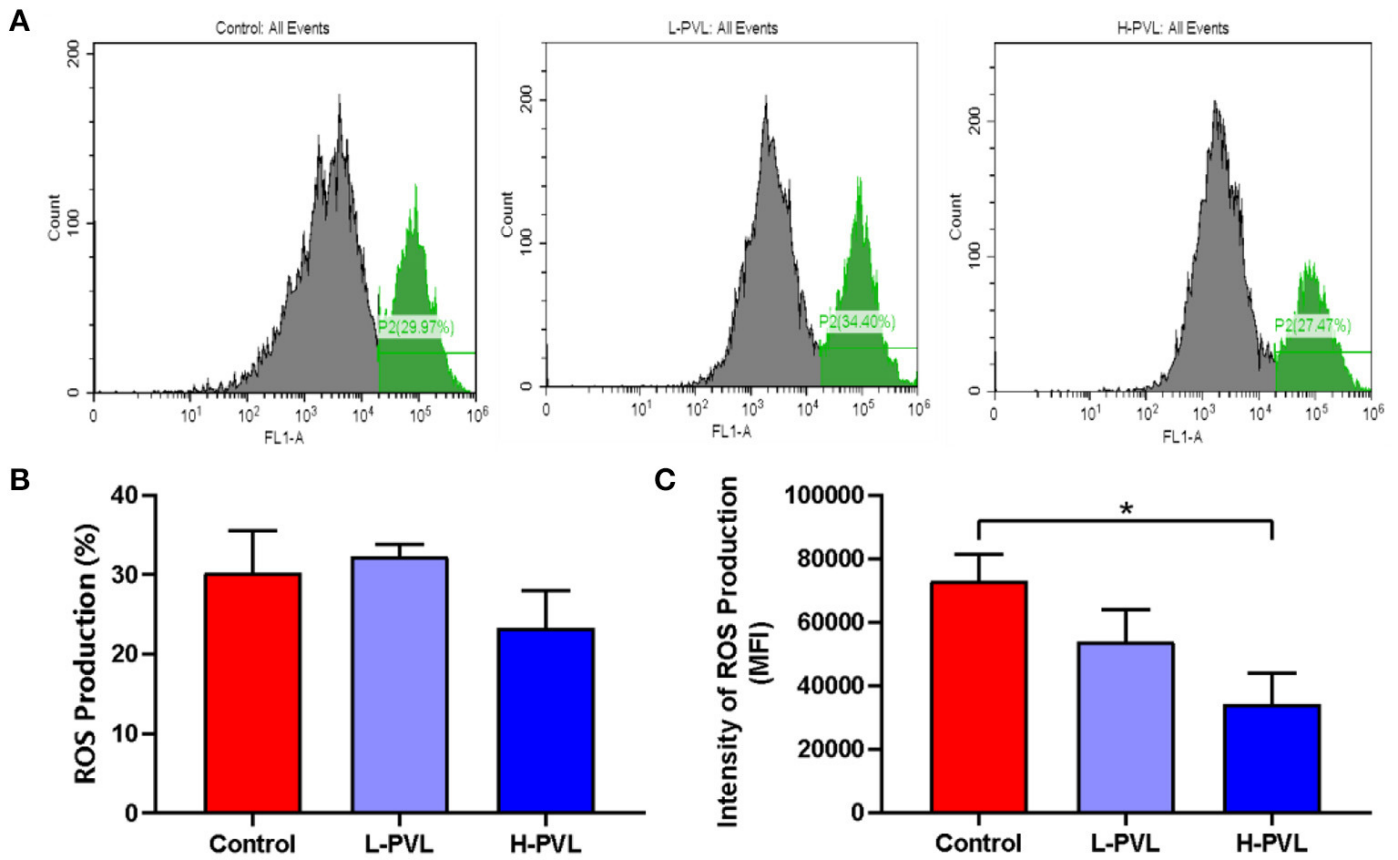
Effect of BLV infection on respiratory burst of PMN. **(A)** Effect of BLV infection on respiratory burst of PMN, **(B)** Effect of BLV infection on ROS production efficiency of PMN, and **(C)** Effect of BLV infection on ROS production in PMN. *P < 0.05 compared to healthy cows.

PMA was also used to stimulate the oxidation function of PMN, and the produced oxides could oxidize dihydrorhodamine 123 into fluorescent rhodamine 123. The MFI of rhodamine 123 was measured using flow cytometry, which could indicate how much oxides were produced by PMNs and reveal the effect of different BLV PVL on the respiratory burst of PMN in cows. The results are shown in Figure 5. There was no significant change in the efficiency of ROS production in cows of the L-PVL and H-PVL groups compared to cows of the healthy group (Figure 5B). Although the intensity of ROS production in cows of the L-PVL group was slightly lower compared to cows of the healthy group, the difference was not statistically significant (*P* > 0.05), whereas the intensity of ROS production in cows of the H-PVL group was significantly lower (*P* < 0.05; Figure 5C).

### Effect of BLV on the Expression of Pro-inflammatory Factors and Adhesion Molecules in PMN

**Figure 6:**
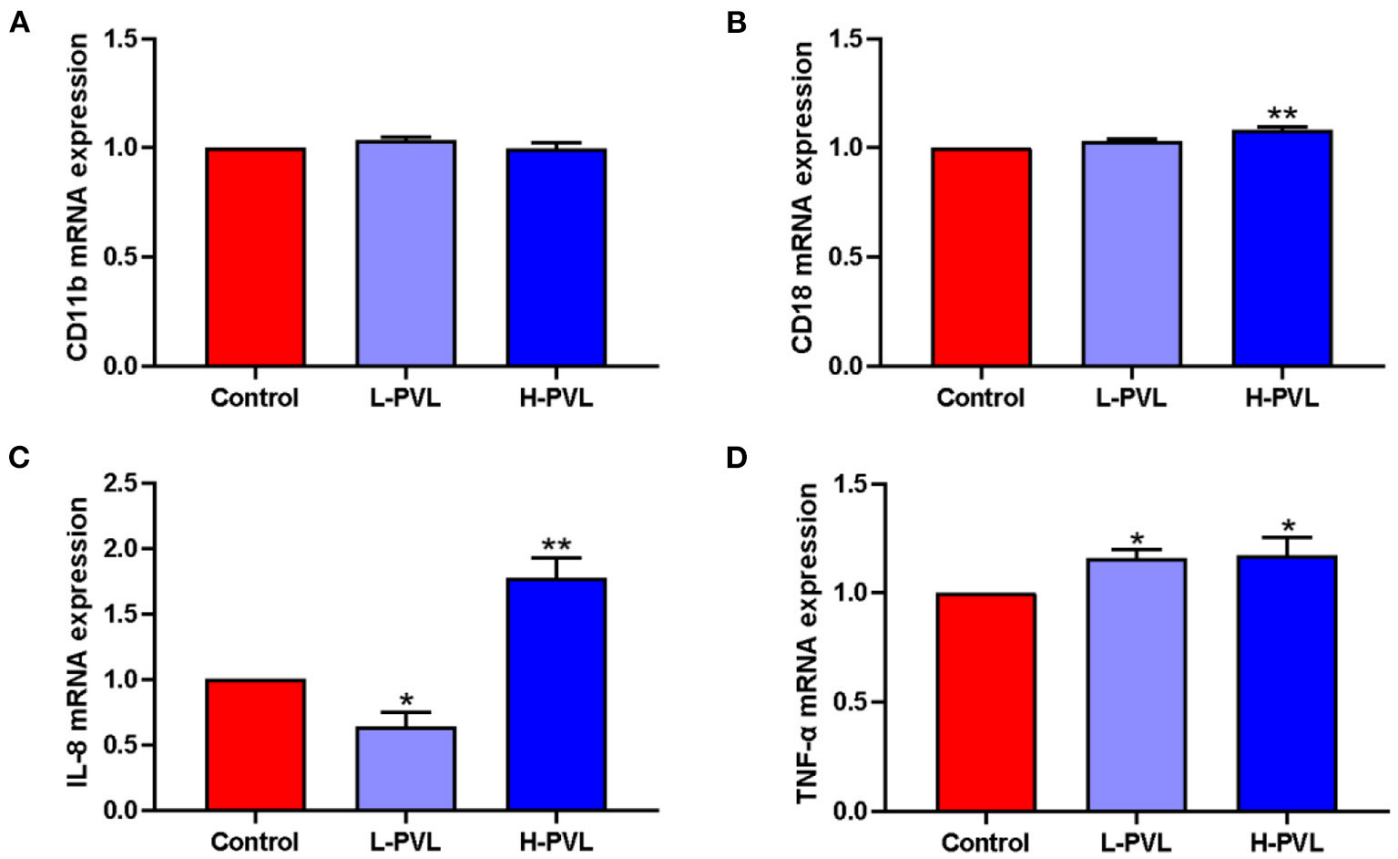
Effect of BLV infection on the mRNA expression of PMN adhesion molecules CD11b, CD18, and inflammatory factors IL-8, TNF-α. **(A)** Effect of BLV infection on CD11b mRNA expression, **(B)** Effect of BLV infection on CD18 mRNA expression, **(C)** effect of BLV infection on IL-8 mRNA expression, and **(D)** Effect of BLV infection on TNF-α mRNA expression. *P < 0.05 compared to healthy cows, **P < 0.01 indicates significant difference compared to healthy cows.

SYBR Green I qRT-PCR was employed to quantify the relevant genes and reveal the effects of different BLV PVL on the mRNA expression of adhesion molecules CD11b and CD18 and pro-inflammatory factors IL-8 and TNF-α in PMN of cows. The results are shown in Figure 6. Although there were changes in the mRNA expression of adhesion molecule CD11b in cows of the L-PVL and H-PVL groups compared to cows of the healthy group, the differences were not statistically significant (*P* > 0.05; Figure 6A). According to Figure 6B, although the mRNA expression of adhesion molecule CD18 was elevated in cows of the L-PVL group compared to cows of the healthy group, the difference was not statistically significant (*P* > 0.05). However, the mRNA expression of adhesion molecule CD18 was significantly elevated (*P* < 0.05) in cows of the H-PVL group than that in healthy group. According to Figure 6C, the mRNA expression of pro-inflammatory factor IL-8 was significantly lower (*P* < 0.05) in cows of the L-PVL group compared to cows of the healthy group. However, the mRNA expression of pro-inflammatory factor IL-8 was highly significantly higher (*P* < 0.01) in cows of the H-PVL group than that in healthy group. According to Figure 6D, the mRNA expression of pro-inflammatory factor TNF-α was significantly higher in the L-PVL and H-PVL groups compared to the healthy group (*P* < 0.05).

## Discussion

PMN is an innate immune cell with a critical role in the immune system. Studies have shown that BLV infection affects the cellular function of both the nonspecific and specific immune systems and even affect some functions of uninfected cells (25). However, research on the effect of BLV infection on the immune function of PMN is scarce. Therefore, the changes in PMN cell activity, chemotaxis, migration, phagocytosis, adhesion, respiratory burst function, and NETs formation in different groups of cows were examined in this experiment. By detecting chemokines CXCL7 and IL-8, it was found that PMN chemokine expression was significantly lower in cows of the L-PVL group compared to cows of the healthy group, but PMN chemokine expression was significantly higher in cows of the H-PVL group. The reason may be that when the BLV PVL is low, the virus remains latent, and only a small number of activated lymphocytes continue to produce new viral particles. In addition, BLV may, like other viruses, achieve immune escape by secreting proteins that interfere with cytokines and chemokines. However, when the latent BLV is activated and proliferates under the stimulation of certain factors, the PMNs are activated and secrete a large number of cytokines to mediate the antiviral immune response.

When stimulated by pathogenic microorganisms, PMNs' expression of cell surface adhesion molecules is altered, contributing to the immune function of PMN. Mac-1 (CD11b/CD18) of the β2 integrin class is a vital adhesion molecule on the surface of PMN. Allergens such as chemokines and cytokines affect the expression of CD11b and CD18 (16). The examination of PMN adhesion and migration function in this study revealed that the expression of CD11b and CD18 was not significantly affected in the L-PVL group compared to the healthy group, the expression of CD11b was not significantly affected in the H-PVL group, the expression of CD18 was significantly higher. BLV infection suppressed PMN transepithelial migration, which may be due to the presence of a specific localized reservoir of Mac-1 adhesion molecules in the dairy PMN, allowing selective mobilization of these molecules in response to external stimuli (26). Mac-1 expression and activation are critical steps in PMN migration to sites of inflammation, and in most cases (24), PMN migration is dependent on CD18. Studies have shown that PBMCs and PMNs in PL cattle express a reduced percentage of CD44, one of the endothelial selectin ligands slowing PMN movement and promoting PMN rolling on the endothelium. CD44 is also considered a PMN phagocytic receptor that effectively mediates the recognition of pathogenic microorganisms (26, 27). The above studies suggest that BLV does not impair PMN migration function exclusively by inhibiting the expression of CD11b and CD18 but instead regulates PMN migration function through multiple factors, such as integrins, fatty acids, and hormones. Some studies suggested that endothelial cell junctions and intravascular environment also affect PMN migration, although the specific mechanisms need further investigation.

PMN removes pathogenic microorganisms in the phagocytosis process, during which ROS is released to kill the invading pathogenic microorganisms. The phagocytosis and oxidative burst activity of PMN are closely related. Therefore, the PMN phagocytosis and respiratory burst function of different groups were successively tested. The results showed that BLV infection had no significant effect on PMN phagocytosis, and the percentages of ROS-producing PMNs were not significantly different among the different groups, but the ROS MFI of the H-PVL group was significantly lower than that of the healthy group. Similarly, Blagitz et al. divided BLV-positive cattle into AL and PL groups based on whether they had persistent lymphocytosis, and detected the production of ROS in their neutrophils. The results showed that the percentage of ROS produced by neutrophils in the PL group was lower than that in the healthy group and AL group (17). It was found in the study of Souza et al. (28) using a smaller number of animals, a decrease in the oxidative burst activity induced by *Escherichia coli* was found, although the oxidative burst activity without any stimulus was not statistical significant, but it is numerically decreased.

Previous studies have shown that IFN-γ has a positive effect on phagocytosis and ROS production in bovine PMNs (29), while BLV infection could reduce T cell IFN-γ expression through PD-1, Tim-3, and LAG3 pathways, which in turn affects PMN phagocytosis and respiratory burst function (30–32). TLRs on the PMN surface is critical for the recognition of bacteria. Studies have shown that the HTLV-1 P30 protein targets the TLR4 signaling pathway, and infections could lead to the downregulation of TLR4 expression on the cell surface (33). Moreover, BLV has a protein with a structure very similar to HTLV-1 P30. Therefore, the observed reduction in ROS MFI may be due to the abnormal expression of CD14 or altered TLR4 signaling pathway caused by BLV infection.

FeLV is a common gammaretrovirus in domestic cats. Hoffmann et al. found that the PMN phagocytosis in FeLV-positive cats was diminished under *E. coli* stimulus (34). However, Wardini et al. (35) tested the PMN phagocytosis in FeLV-positive cats using Leishmania promastigotes as stimulus and found no significant effect of FeLV infection on PMN phagocytosis in cats. In contrast to the results of other scholars, the results of this experiment showed no significant effect of BLV infection on PMN phagocytosis of *S. aureus*. This inconsistency may be attributed to the different methodologies, the different pathogenic microorganisms used to assess PMN phagocytosis or the different sample sizes.

PMN facilitates the clearance of pathogenic microorganisms by producing NETs that trap and kill bacteria and fungi. Recent studies have shown that NETs help clear viruses or stop local transmission of viruses within host tissues. However, NETs have favorable and unfavorable effects during the antiviral response. On the one hand, NETs have the basic effective mechanism to capture viruses. On the other hand, the release and aggregation of NETs could cause tissue damage. The interactions between NETs and platelets have been shown to cause endothelial damage in *E. coli*-induced infections (24). This study showed that the release of NETs in the L-PVL group was not significantly different compared to those in the healthy group, but the release of NETs in the H-PVL group was significantly higher than that in the healthy group. The reason may be that, as previously described, when the BLV PVL is low, the virus is still latent, and the number of viral particles replicated and released into circulation is not significant. Once the virus is massively replicated and released into circulation, PMNs produce large numbers of NETs through a mechanism similar to the clearance of HIV-1 or other pathways and participate in the anti-BLV immune response. In addition, the experiment showed that the expression of TNF-α and IL-8, factors inducing the release of NETs, was significantly higher in the H-PVL group than that in the healthy group. Altered PMN immune function was also detected in other viral infections, particularly HIV-1 and HTLV-1. Studies have shown that FeLV infection caused spontaneous upregulation of MPO secretion and enhanced MPO activity in feline PMN without other stimuli, resulting in a significant increase in the release of NETs (36). Therefore, this progressive viral infection may induce chronic activation of PMN and contribute to a series of immune responses in PMN.

The effects of various viruses on innate and adaptive immunity predispose animals to different types of co-infection or mixed infections and may increase the severity of the infections. The effects of BLV are often underestimated as EBL is a chronic disease with no apparent clinical symptoms and a low lethality. Our findings indicated that BLV infection could affect the immune function of PMNs in dairy cows, and the effects were closely related to BLV PVL. However, the specific mechanisms by which BLV infection affects PMN immune function and PMN's relationship with other bacterial diseases are not clear, which require further research. Nevertheless, cows with high BLV PVL are more likely to transmit BLV and develop mastitis than healthy cows or cows with low BLV PVL (10, 11). Attention should be paid to cows with high BLV PVL, and culling them would reduce BLV transmission and infections in the herd (21, 36), which could constitute economically viable BLV eradication programs and strategies.

## Data Availability Statement

The raw data supporting the conclusions of this article will be made available by the authors, without undue reservation.

## Ethics Statement

The Laboratory Animal Ethics Committee College of Animal Science and Veterinary Medicine, Heilongjiang Bayi Agricultural University (Daqing, China) approved the study protocol (No. BAYU20191121).

## Author Contributions

RW and JW contributed to the conception of the study. HW contributed significantly to analysis and manuscript preparation. GL performed the data analyses and wrote the manuscript. SL helped perform the analysis with constructive discussions. All authors contributed to the article and approved the submitted version.

## Funding

This study was supported by grants from the National Natural Science Foundation of China (Nos. 31972747 and 32002247) and Natural Science Foundation of Heilongjiang Province of China (Nos. C2018043 and YQ2019C014).

## Conflict of Interest

The authors declare that the research was conducted in the absence of any commercial or financial relationships that could be construed as a potential conflict of interest.

## Publisher's Note

All claims expressed in this article are solely those of the authors and do not necessarily represent those of their affiliated organizations, or those of the publisher, the editors and the reviewers. Any product that may be evaluated in this article, or claim that may be made by its manufacturer, is not guaranteed or endorsed by the publisher.
